# A Natural Human-Drone Embodied Interface: Empirical Comparison With a Traditional Interface

**DOI:** 10.3389/fnbot.2022.898859

**Published:** 2022-10-14

**Authors:** Marina Di Vincenzo, Francesco Palini, Maria De Marsico, Anna M. Borghi, Gianluca Baldassarre

**Affiliations:** ^1^AS-AI - Advanced School of AI, Rome, Italy; ^2^Department of Computer Science, Sapienza University of Rome, Rome, Italy; ^3^Department of Dynamic and Clinical Psychology and Health Studies, Sapienza University of Rome, Rome, Italy; ^4^Institute of Cognitive Sciences and Technologies, National Research Council, Rome, Italy; ^5^AI2Life Srl Innovative Startup, Spin-Off of ISTC-CNR, Rome, Italy

**Keywords:** human-drone interface, human-machine interface, flexible software dAIsy, human performance, exploration and accuracy tasks, embodiment and virtual experience questionnaires

## Abstract

Despite the importance of usability in human-machine interaction (HMI), most commonly used devices are not usable by all potential users. In particular, users with low or null technological experience, or with special needs, require carefully designed systems and easy-to-use interfaces supporting recognition over recall. To this purpose, Natural User Interfaces (NUIs) represent an effective strategy as the user's learning is facilitated by features of the interface that mimic the human “natural” sensorimotor embodied interactions with the environment. This paper compares the usability of a new NUI (based on an eye-tracker and hand gesture recognition) with a traditional interface (keyboard) for the distal control of a simulated drone flying in a virtual environment. The whole interface relies on “dAIsy”, a new software allowing the flexible use of different input devices and the control of different robotic platforms. The 59 users involved in the study were required to complete two tasks with each interface, while their performance was recorded: (a) exploration: detecting trees embedded in an urban environment; (b) accuracy: guiding the drone as accurately and fast as possible along a predefined track. Then they were administered questionnaires regarding the user's background, the perceived embodiment of the device, and the perceived quality of the virtual experience while either using the NUI or the traditional interface. The results appear controversial and call for further investigation: (a) contrary to our hypothesis, the specific NUI used led to lower performance than the traditional interface; (b) however, the NUI was evaluated as more natural and embodied. The final part of the paper discusses the possible causes underlying these results that suggest possible future improvements of the NUI.

## Introduction

The best technology is the one that cannot be seen because it is so simple to use that it becomes “invisible” (Norman, 1998). The design and implementation of Natural User Interfaces, or NUIs, is nurtured by technologies intended to replace more traditional GUIs (Graphical User Interfaces), which in turn previously replaced CLI (Command Line Interfaces). One of the pioneering researchers and visionary inventors in this field was Steve Mann, who in the 1990s conceived and developed examples of natural interaction with computers, or “digital agents”, referring to these as “natural user interfaces”. In Mann's view, these are implemented by wearable computing (Mann, 1998). However, in the HCI community the expression has passed to indicate what Turk (2001) defines as “perceptual interfaces”, whose aim is “*to make human-computer interaction more like how people interact with each other and with the world.”* As the word itself suggests, NUIs are closer to people's common ways to communicate and interact with the environment inasmuch they entail actions that come naturally to human users. The user does not act via artificial communication devices such as the keyboard or mouse but rather communicates directly through body expressions, such as voice, gestures, gaze, and behavior (Rautaray and Agrawal, 2015). It is important to underline that the term “natural” is used to describe a property of the technology that is external to the product itself: thus, the “natural” element of a NUI does not refer to the interface itself, but to the way users interact and perceive it (Norman, 1988). Of course, the interface technology is intended to support such interaction styles. The main objective of the introduction of NUIs is to facilitate communication between humans and machines and improve technology usability (Rauterberg, 1999). Being based on the users' native capabilities, NUIs require little or no prior knowledge and learning to be used. By design, they imply that recognition is privileged with respect to recall for triggering actions and detecting their effects. Regarding the complex and sometimes controversial relationships between recognition and recall, the interested readers may refer to the experiments in Hanawalt and Tarr (1961) and to the usability-related considerations in Hoyer and Brown (1990). The design of NUIs gives the user the feeling of immediate and continuous success, while interaction is felt as easy and intuitive because it is based on reality [RBI—Reality-Based (Wigdor and Wixon, 2011)]. In addition, NUIs tend to become *invisible*, and this takes us to the paradigm of *Embodied User Interfaces*. In this respect, the seminal work in Fishkin et al. (1998) presents and defines this paradigm together with a set of design principles to guide design. The technology at that time somehow limited the feasibility of the most advanced embodied solutions. This has changed with the spread of increasingly sophisticated and affordable devices in the customer market. Nowadays, these allow amazingly immersive virtual reality interfaces (Serra et al., 2020). Embodiment can be considered as one of the desirable features of a NUI.

The research and design of suitable interfaces for these tools, which are usable and affordable by everyone, could have substantial effects on the quality of life of people in different contexts of everyday life (Turunen et al., 2009). Being intrinsically multimodal, NUIs also have great potential in the field of assistive technology for their enhanced usability and potential to overcome physical boundaries. An example of a completely voice-driven dictation prototype can be found in De Marsico and Mattei (2021) where the sight-impaired user dictates as it would do with a human secretary, without any need to acquire visual awareness of the position in the text. Apart from “serious” applications, multimodality and natural interaction also play a relevant role in the amusement field.

The opportunities for interaction between humans and drones are increasing and NUIs have strong potential for drone control. Several studies in the literature report the use of natural interfaces to pilot drones. Among the different ways of interaction, we find gestures. A summary of recent research is reported in the next section.

A key characteristic of the device tested here “embodiment level”. To clarify what this is, it is worth briefly referring to the current cognitive science and neuroscience literature on this topic. With the term “embodiment”, we refer to the sense of one's body, a topic which has been the object of many investigations in recent years (Longo et al., 2008). Studies have highlighted how the experience of possessing a body is crucial for our sense of conscious self (Seth and Tsakiris, 2018). These show that we ascribe relevance to changing perceptual experiences thanks to their relationship with our subjective bodily experience. We ascribe perceptions and sensations to ourselves in virtue of the capability to distinguish our body from the body of others (Aglioti and Candidi, 2011). In representing our body, we integrate inputs of different nature (visual, tactile, and proprioceptive) to maintain *multisensory* coherence. Importantly, our sense of body is not static but can change. Various experimental studies using illusion paradigms with multisensory manipulations have investigated to what extent the body sense is malleable and demonstrated that it could be stretched and extended (Maravita and Iriki, 2004; Borghi and Cimatti, 2010; Borghi et al., 2013; Scorolli et al., 2016). An example is represented by effects such as the so-called facial enhancement effect. Participants observe someone else's face being touched, while receiving tactile stimulation on their own face; this increases the feeling of similarity with the other, blurring the self-other distinction (Tajadura-Jiménez et al., 2012; Fini et al., 2013). Crucially for the present work, influential studies have also shown that the sense of body ownership goes beyond the actual possession of body parts. Much evidence has been collected with the rubber hand illusion (RHI) (Botvinick and Cohen, 1998), in which participants embody a fake hand, provided that it is located where their own hand should be, and it is touched synchronously with their hand. The illusion's strength decreases with a wooden hand or when the hand is presented in a biomechanically implausible posture. Instead, the illusion is maintained when the hand has a different skin color and is enlarged, thus showing that the perceptual similarity to the participant's hand is not essential (for a review, see Ratcliffe and Newport, 2017). The discovery that external objects can be embodied and considered as extensions of our own body has had a variety of applications. In our study, testing to what extent the device increments the sense of embodiment for the user is crucial since a higher embodiment might lead to perceiving the interface's use as more natural and smooth.

This work investigates if and how NUIs could improve users' performance and give a greater sense of easy embodied use with respect to more standard interfaces allowing to remotely control a drone. The particular NUIs we use for this purpose are based on a system formed by an eye-tracking device and a gesture recognition system (Natural interface, also indicated in the following as “N”). This NUI is compared to a traditional keyboard interface (Traditional interface, also indicated with “T”). These two interfaces are compared as employed to control a simulated flying drone to perform two tasks: an *exploration task* (counting trees hidden in an urban environment) and an *accuracy task* (following a track as accurately and fast as possible) in virtual reality scenarios. The two interfaces were compared using a within-participants experimental design, where all participants took part in every condition and answered the related administered questionnaires. These questionnaires consider the user's background and assess the perceived embodiment and the perceived quality of the virtual experience in both conditions. In particular, the experiments aimed at testing three hypotheses: (1) *Performance*: the use of the natural interface to control the drone gives the user better control over the device and so leads to a better performance with respect to the traditional interface; (2) *Embodiment*: the use of the natural interface to control the drone gives the user a higher sense of embodiment with respect to the traditional interface; (3) *Correlation*: performance and level of embodiment are positively correlated.

The rest of the paper is organized as follows. The Section Related Work on Traditional and Natural Interaction-Based Drone Control and Its Evaluation summarizes both different classes of approaches to remote drone control and the most popular evaluation tools. The Section Methods presents a new software platform, called dAIsy, that can be used to implement different interfaces by allowing an easy plug of different input, such as the eye-tracker, the hand-gesture, and the keyboard used here, and the control of different robot devices, such as the drone used here. The Section Methods also introduces the experimental protocol used to test the interfaces. The Section Results presents the results. A Section Discussion follows that discusses and interprets the results. The Section Conclusion summarizes the results and discusses possible future work based on the results.

## Related Work on Traditional and Natural Interaction-Based Drone Control and Its Evaluation

In the last years, UAV technology has moved from specialized applications, e.g., military and advanced video surveillance, to everyday normal uses such as amusement. Even though unmanned aerial vehicles (UAVs) do not carry pilots on board, they still require operators planning and controlling critical functions. These operators need also to interpret the sensor information provided by these platforms. This applies to all classes of systems, from true small planes (the smaller portable systems nowadays can reach miniaturized dimensions of 5 cm or less; Tu et al., 2021), as well as to UAV swarms (Campion et al., 2018). As a consequence of the increasing popularity, especially in amusement applications, drone control interfaces have evolved. These initially required highly specialization, needing operators' long and accurate training, for example for real-time reconnaissance and control in critical actions during military operations. Instead, now we have more friendly interfaces exploiting natural interactions and enhanced by improved self-stabilization drone mechanisms. Many recent works deal with drone communication protocols (Hassija et al., 2021), with drone detection in military and security-related contexts (Chiper et al., 2022), or with specific drone applications, either military or civil (e.g., Roldán-Gómez et al., 2021), or for entertainment (Kim et al., 2018).

This paper focuses on UAV remote control interface. Drone driving is a special case of vehicle teleoperation (Fong and Thorpe, 2001). It is possible to identify four classes of drone driving equipment: (a) keyboard and mouse; (b) gamepad, joystick or physical knobs; (c) virtual joystick or haptic/touch-based; (d) natural interaction-supporting devices. The first category is typically combined with an element of the second one and is used in ground control stations for both military and civil applications (Haque et al., 2017). The second category, some elements of which have been also compared (Rupp et al., 2013), is very frequently used in entertainment (Kim et al., 2018). A customer-level instance of the third category involving a virtual joystick is provided by Parrot with the AR.FreeFlight 2 application interface (Parrot, 2012); a second instance regards a prototype of multitouch drone control (Kang et al., 2018). The fourth and last category of interfaces is the one considered in this paper (Mirri et al., 2019) and so discussed more extensively.

The control methods of the fourth category rely on perceptual and motor enabling technologies expanding the taxonomy proposed by Karam and Schraefel (2005) for gestural interaction (including video, audio and remote sensing) by relying on human natural output channels such as gestures, speech, and gaze to issue commands via video and audio channels. Gesture-based commands represent the most popular approach. In a study by Cauchard et al. (2015), users were asked to define their preferred gestures to control the drone. The results showed that 90% of the participants stated that they felt in control, and 95% felt that it was natural to interact with the drone. An example of drone driving based on hand gestures, captured by a smartphone communicating with the drone, is presented in De Marsico and Spagnoli (2019). Further studies investigated the control of drones by eye-tracking in a real environment (Alapetite et al., 2012; Yuan et al., 2019), possibly complemented by keyboard shortcuts (Zhou et al., 2022). Others have instead used multimodal controls, including voice, gesture, and full-body control. An example of body-driven control is represented by an upper body soft exoskeleton for immersive drone control (Rognon et al., 2018). The most advanced frontier is to use a brain-computer interface (BCI; Nourmohammadi et al., 2018). Most of these studies have shown that the use of NUIs for drone control is feasible and involves a greater sense of communication between the drone and the human (Fernandez et al., 2016). But, of course, the most sophisticated control equipment, at least at present, is devoted to critical applications, for example military ones, and is far from being widely available on the market. According to this, the present proposal exploits a multimodal approach with gaze and gesture tracking which presently could be widely employed in customer-level devices.

Regarding system evaluation, it is necessary to distinguish pure task execution accuracy from user's satisfaction. Regarding the pure driving performance, popular measures involve measures of the distance between the drone center and waypoints set along the ideal trajectory to follow (Cherpillod et al., 2019). Performance per participant is for example computed as the root mean square (RMS) of this distance averaged over the waypoints of a task. User-centered design issues are discussed by Mouloua et al. (2003). At a more general level, it is interesting to consider a recent work on the evaluation of an embodied control interface (Bekta et al., 2021). This work identifies three categories of key concepts underlying the evaluation, and each category calls for suitable evaluation tools. The first category is *Control*, which includes statements referring to the effects of the control interface on the controlled system. Related keywords are “usability”, “control”, “mapping”, “reaction”, and “responsiveness”. In the cited experiments, participants, feedback on Control is scored with the results of the System Usability Scale (SUS) questionnaire (Brooke, 1996). The second category is *Task*, which regards the effects of the control interface on the experimental task. The authors identify as keywords “difficulty”, “attention”, “concentration”, “stress”, and “training”; they evaluate the outcome in terms of performance measures including the time required by the task, the task execution accuracy, and the NASA Task Load Index (TLX) questionnaire (Hart and Staveland, 1988). The last category is *User*, which is related to the effect of the control interface on user experience. This last category is the one closer to a measure of embodiment. The related keywords are “comfort”, “intuitiveness”, “naturalness”, “experience”, “nausea”, “sickness”, “enjoyment”, “presence”, and “involvement”. The authors measure this category using the Presence Questionnaire (PQ; Witmer and Singer, 1998), the Simulator Sickness Questionnaire (Kennedy et al., 1993), and body sway measurements. The latter two are better suited for fully immersive applications, like simulators, not considered here. The detailed consideration of the cited study highlights some basic points related to the evaluation of prototypes entailing embodied control, either using fully immersive setups or natural interaction: (a) it is necessary to consider different complementary aspects that relate to both the specific task performance and the user comfort; (b) the different nature of the performance/usability measures suggest using the composition of specifically designed evaluation tools in a kind of plug & play strategy; (c) traditional questionnaires employed in usability and user experience evaluations can be combined to assess different aspects in complex applications. The evaluation of the presented proposal stems from these considerations.

## Methods

### dAIsy

The software platform created to implement both the NUI and the traditional interface is called “*dAIsy—Device Alternative Interaction System'*'. dAIsy is based on a multithreaded software written in Python that allows several commands based on different input devices to be executed simultaneously on the same controlled device. dAIsy consists of several modules that interact with each other, in particular:

- *Controller*: the module coordinating all the other components described below.
- *Device*: this module represents the controlled device (the drone here or another robot) that performs actions in the environment.
- *View*: this module shows on a monitor the video streamed by the controlled device, recorded through its onboard camera (e.g., the camera on the drone, as here, or of another controlled robot).
- *Listener*: a module representing an interface device (here the eye tracker, hand-gesture recognition, keyboard) which “listens” for the user's inputs; one or more listeners can be used.
- *Mapper*: the module that maps the user input to the corresponding actions to be performed by the device (the mapping changes according to the chosen listeners and devices).
- *Detector*: a module that detects and locates the objects of interest in the scene based on computer vision techniques; this module has not been used in the experiments illustrated here.

**Figure 1:**
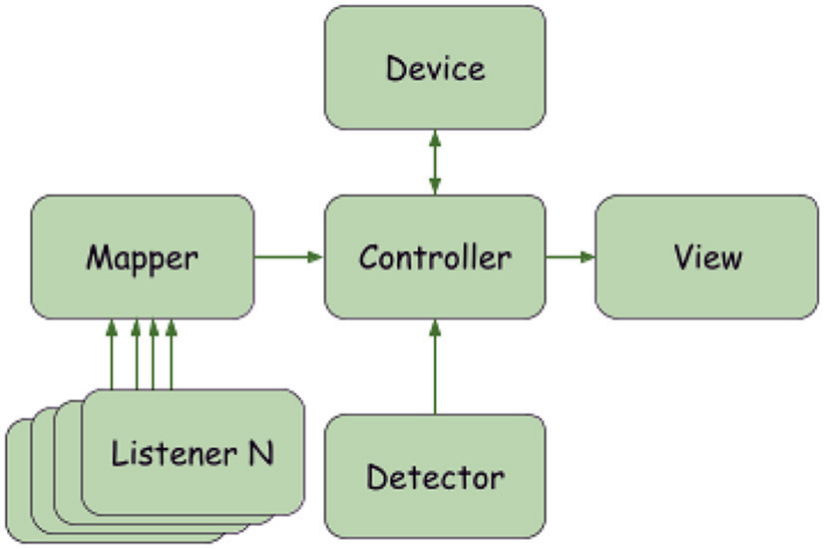
A Block diagram illustrating the functioning and main components of the dAIsy software.

One of the key features of dAIsy is flexibility. In particular, the connection of new input and controlled devices is based on the modification of a configuration file. Figure 1 presents the interactions among the system's components. Each listener is able to trigger commands to the device through the Controller module. As described in the next section, each Listener checks which command to execute based on the event triggered, by using the Mapper module. The Controller periodically takes new video frames from the device (this is the reason for the arrow from the Device to the Controller in the figure) and updates the View. The Detector module is used each time a new frame is taken from the drone by the Controller.

All modules can be easily modified and replaced. Three listeners were used in the study: Keyboard Listener, Gaze Listener, and Hand Listener. In particular, condition N used all three Listeners, whereas condition T only the Keyboard Listener. In the following paragraphs, the Device and Listeners implementations are described more in depth.

#### Mapper

The Mapper module is used to logically separate the commands, specific to the Device, from the events, specific to the Listeners. Each event is mapped to a command id, which is interpreted on the Device side. For example, the event KEY_T (pressing the key T on the keyboard) has the command id 0, interpreted by the drone as “take off”. The event VOICE_TAKE_OFF (associated with the Voice Listener) has the same command id 0, so that both the Listeners can be used to take off the drone. In this way the Listeners have only to manage their events without thinking about the effects and, if needed, the dAIsy user can choose to associate the VOICE_TAKE_OFF event with a different command by simply changing the command id.

#### Drone Device

Nowadays, several companies are developing and selling drones making available an SDK (Software Development Kit) for commercial and research purposes. For our purposes, the most suitable library turned out to be the Olympe SDK (Olympe, 2022, https://developer.parrot.com/docs/olympe), developed by Parrot. The Olympe library, written in Python, easily allows establishing a connection with the drone, getting telemetric information and sending the piloting commands. The Device abstraction made available by dAIsy allows mapping the inputs obtained by the interfaces to coherent values that can be sent as drone command parameters. Moreover, the abstraction allows getting each frame from the device and sending it to the monitor. Our experiments used a simulated version of the real Parrot drone, which was connected to the computer through an IP address. The same kind of communication is defined for the real drone and dAIsy directly supports it: this switch requires only to change the configuration parameter “device” from “drone.SimulatedDrone” to “drone.PhysicalDrone”. The commands are sent to the drone by using the function *piloting_pcmd*, provided by the Olympe library, at a rate of 20 Hz. The function arguments are roll, pitch, yaw angles and gaz (throttle), expressed as a signed percentage, thus assuming values in [−100, 100].

#### Keyboard Listener

**Figure 2:**
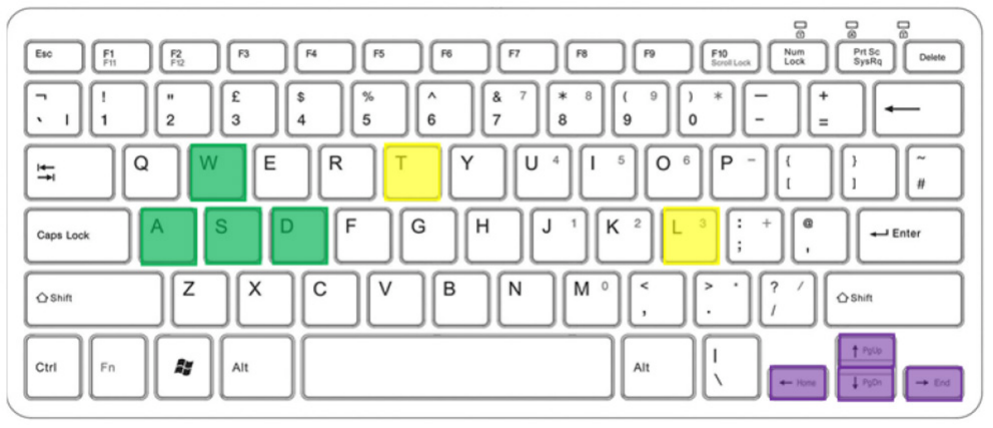
Keyboard keys used to control the drone: in yellow T and L for take-off and landing; in purple the arrows to direct the drone's gaze; in green the WASD keys for movement.

The implemented Keyboard Listener is based on the *pynput* library. The library allows detecting an event when a key is pressed or released. Some keys were associated with the commands to be issued to the drone, in particular (Figure 2):

- “t”: take-off;
- “l”: landing;
- “arrow up”, “arrow down”: to regulate the actuated pan and tilt of the camera;
- “arrow left”, “arrow down”: to turn the drone anticlockwise and clockwise;
- “w”, “a”, “s”, “d”: to move the drone respectively forward, leftward, backward, and rightward.

#### Gaze Listener

**Figure 3:**
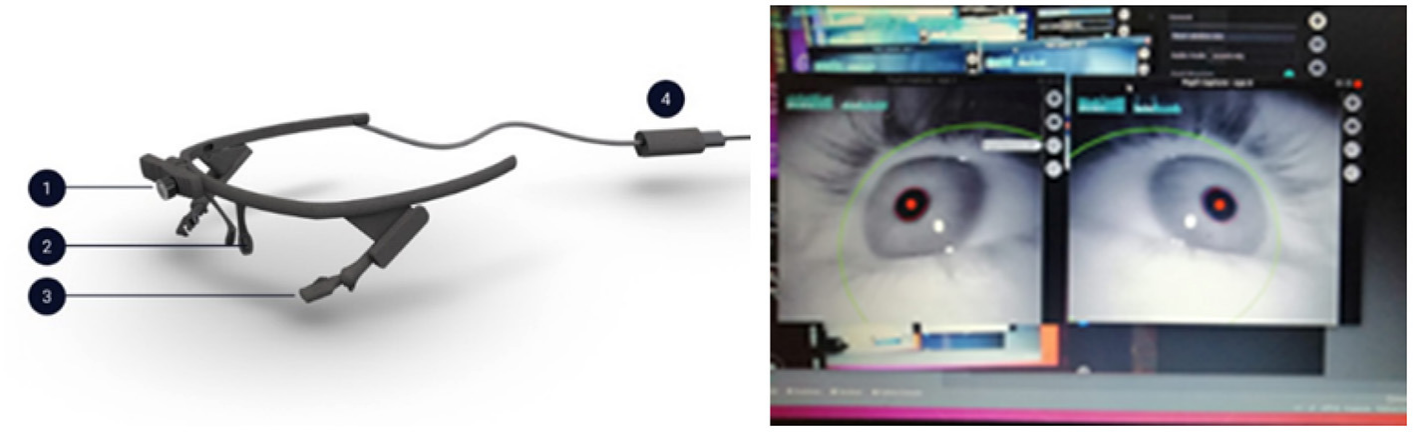
**Left**: Pupil Core goggles. 1: front camera; 2 and 3: eye-oriented cameras; 4: cable connecting the goggles to the computer. **Right**: green framing around the eye image and red dot on the pupil image indicating that all cameras have been positioned correctly.

The Gaze Listener interfaces the system with the user's gaze by using a *Pupil Core* eye-tracker (Pupil Core, 2022, https://pupil-labs.com/products/core/). This is formed by wearable lensless goggles equipped with 1 front camera and 2 eye-oriented cameras (Figure 3). During the experiment, the image captured by the onboard drone camera is shown on a screen located in front of the user: in this way, the user can see, through the drone's camera, the scene explored by the drone itself. The Listener uses the Pupil Core open-source eye-tracking library. The library uses state-of-the-art Computer Vision algorithms for real-time pupil detection and tracking based on the 2 eye-oriented cameras, while the pupil positions are mapped to the space (gaze mapping) by involving the front camera and by using a transfer function (Kassner et al., 2014). Moreover, each detection message sent by the pupil to our system contains a “confidence” value ranging in [0, 1], indicating the confidence about the quality of the detection. We choose to filter messages with confidence of at least 0.8. The Pupil Core software performs the detection of the position of the user's gaze, and its mapping to a specific point within the image captured by the Pupil Core front camera and corresponding to the scene that the user is looking at on the screen. On this basis, the Listener automatically issues pan-tilt commands to the drone's camera and anticlockwise/clockwise rotation commands to the drone so that the point gazed by the user on the screen image (e.g., an object) moves to the center of the image observed by the user on the screen. This mimics what naturally happens to the retinal image when a human gazes at a certain object.

Before its use, the Pupil Core eye cameras are adjusted so as to be adapted to the face configuration of the user. After this is done, an outer green circle and a red dot projected on the center of the pupil image as reported on a monitoring screen indicate that the eye is correctly framed by the system (Figure 3).

Once the cameras are correctly adjusted, they need to be calibrated through a procedure requiring about a minute of fixation of the user on some landmark dots projected on a white background in the screen. During the calibration, the user orients the face toward the screen so that the Pupil Core camera can see the whole image projected on the screen itself. This allows the Pupil Core to record the correspondence between the scene observed by the user (screen image) and the pupils' position corresponding to a certain gaze. After these operations, the use of the Pupil Core to control the drone's rotation and camera pan-tilt is simple as it requires the user to just look at any point of interest on the screen, and the drone will move to look at the same location so that the fixated point moves toward the center of the image on the screen.

Once dAIsy software starts, the user can see on the monitor what the drone is “seeing” through its camera. In addition to the camera frames sent by the drone, dAIsy adds on the corners four AirTags (Surface Tracking, https://docs.pupil-labs.com/core/software/pupil-capture/#surface-tracking) that the Pupil Core goggles use to identify the boundaries of the relevant surface (i.e., the monitor), so that it can map the gaze correctly. The result of the gaze detection, returned by the Pupil Core messages, is a pair of coordinates (x,y) with values in [0, 1], having [0.5, 0.5] as the center of the surface. In order to map the goggles' output to device command inputs, we implemented a PID (Proportional Integral Derivative) controller. After controllability tests we found that values (P = 140, I = 0, D = 0), assigned to the controller, result in a smooth and natural control of the drone.

It is fundamental that the user maintains the head still in order to allow the Pupil Core to identify all the four AirTags. If one or more AirTags are out of sight of the external camera, the goggles cannot detect the surface on which the gaze should be mapped and no command is sent to the Device. In addition, when the gaze detection is inaccurate for any reason (e.g., the user blinks), no command is sent since the “confidence” of the detection is filtered, as stated above, at 0.8. The eye-oriented cameras are able to sample the frames at 200fps with a resolution of 192 x 192 pixels. The front camera is able to sample the frames at 60fps with a resolution of 720p. Pupil Core is able to compute the gaze with an accuracy of 0.6 degrees.

#### Hand Listener

**Figure 4:**
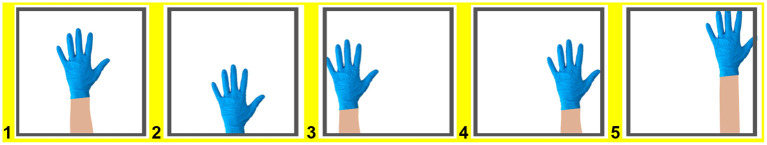
Hand Listener: commands issued to the drone via hand gestures. The offset of the hand with respect to the center indicates the planar direction toward which the drone has to move. The five hand position examples in the figure indicate that the drone should move respectively: forward (1), backward (2), rightward (3), leftward (4), and forward-leftward (5).

The hand control is based on a custom color-based blob detection algorithm written in Python, which exploits one of the most used libraries for computer vision, *OpenCV*. A webcam is oriented toward the user's hand, and the software identifies the hand itself as the “blob” to consider. In order to improve the process accuracy, the user wore a blue glove and the blue-color blob was used by the software as the stimulus to be tracked. At present, the system's ability to directly exploit bare hand images was out of the scope of this work. We plan is to enhance this aspext in future work. The software identifies the position of the hand within an imaginary square in the camera field of view, and this is used to issue commands to the drone. In particular, the commands control the planar movements of the drone based on the offset of the hand position (center of the colored blob) with respect to the central point of the square area (Figure 4). This also allows the regulation of the speed of the drone's movement in the desired direction based on the size of the offset.

The Hand Listener command rate depends on the webcam hardware specifications, considering that the frame rate is lower because of the blob detection algorithm. The webcam used during the experiments has a frame rate of 30fps with a resolution of 1080p.

### Participants

Fifty-nine healthy participants (19 females, 40 males) took part in the study. The mean age of the participants was 27.3 years (SD = 5.8; range: 18–58 years). People with visual impairments or left-handed were excluded from the study. The reason for the first type of exclusion is that visual impairment intrinsically prevents such users from exploiting the vision-based module. The reason for the second type of exclusion is merely related to the present setup, where the camera captures the hand gestures points of the right hand of the user, and the software module recognizes right hand configurations. This can be trivially extended in the future by using two cameras and creating a mirrored version of the software. However, at the moment the aim of the study was just to compare N and T interfaces.

Participation was voluntary, and before running the experiment each participant had to sign an informed consent form. The form and the experimental protocol was approved by the *Ethics Committee* of the *Department of Dynamic and Clinical Psychology, and Health Studies* of *Sapienza University of Rome*.

Using a within-participants experimental design, participants performed the two tasks (Exploration, Accuracy), in the two conditions (traditional “T”, natural “N”). The sequence of the tasks was always the same. Instead, the order of the conditions was alternated: 30 participants experienced the T condition in the first session and the N condition in the second session, whereas 29 participants first experienced the N condition and then the T condition.

### Experimental Setup

**Figure 5:**
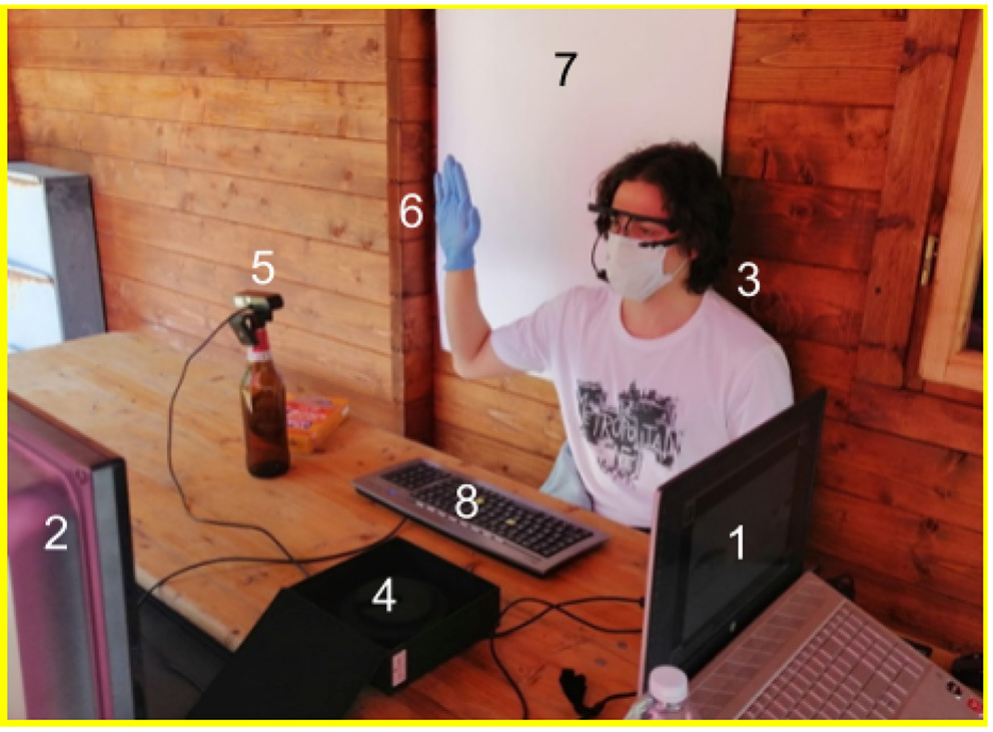
Setup of the experiment involving: (1) Experimenter's computer used to control the whole system through dAIsy; (2) Screen where the streaming images seen by the drone are shown to the participant; (3) Experiment participant wearing the eye-tracking Pupil Core goggles for gaze identification; (4) container used to host the fragile Pupil Core goggles during intervals between different experimental sessions; (5) Webcam to detect hand gesture; (6) Hand with glove for gesture recognition; (7) White background to facilitate hand gesture recognition; (8) Keyboard of the traditional interface.

The participant was set with the back against a wall (Figure 5). A 46-inch screen showing the streaming images seen by the drone was located 120 cm away from the participant who could see them. The keyboard of the traditional interface was located on a table in front of the participant. The webcam for the hand-gesture recognition NUI was located in front of the participant's right hand, and a white panel covering the whole view of the webcam was located on the wall behind the participant for enhancing color contrast. The Pupil Core goggles were worn by the user as normal eyeglasses. The experimenter sat on the left side of the table and operated the computer controlling the whole system through dAIsy.

### Virtual Environment and Tasks

**Figure 6:**
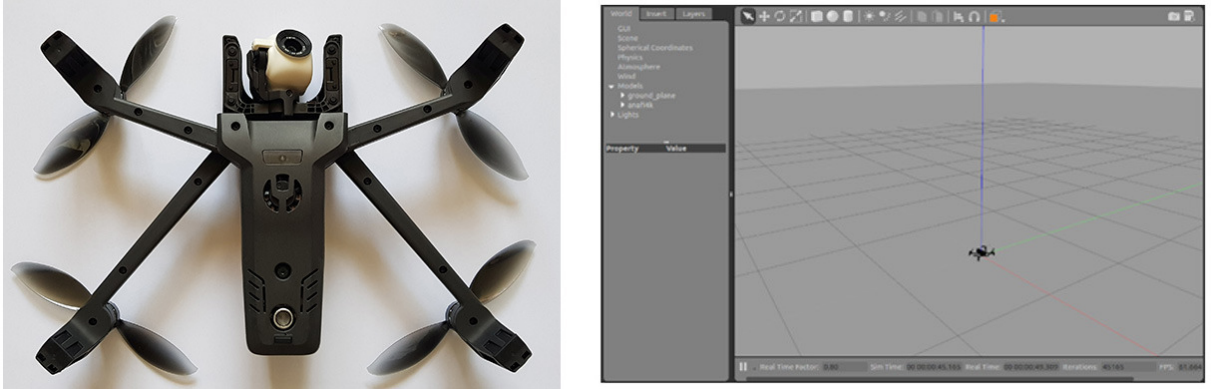
**Left**: *Anafi Parrot* drone. **Right**: a snapshot of the rendering of the *Parrot Sphinx* simulator used in the experiments.

Here we used dAIsy to control the *Anafi Parrot* drone (Anafi Parrot, https://www.parrot.com/en/drones/anafi) (Figure 6) as a device. This drone comes with the *Parrot Sphinx* simulator system (version 1.9) that we used to create the virtual environment considered for the experiments (Figure 6). In the virtual environment, the view was in first person as the users explored the scene through the drone's camera. The video shown to the users reached a frame rate of a common video, near 30 FPS (frames per second).

**Figure 7:**
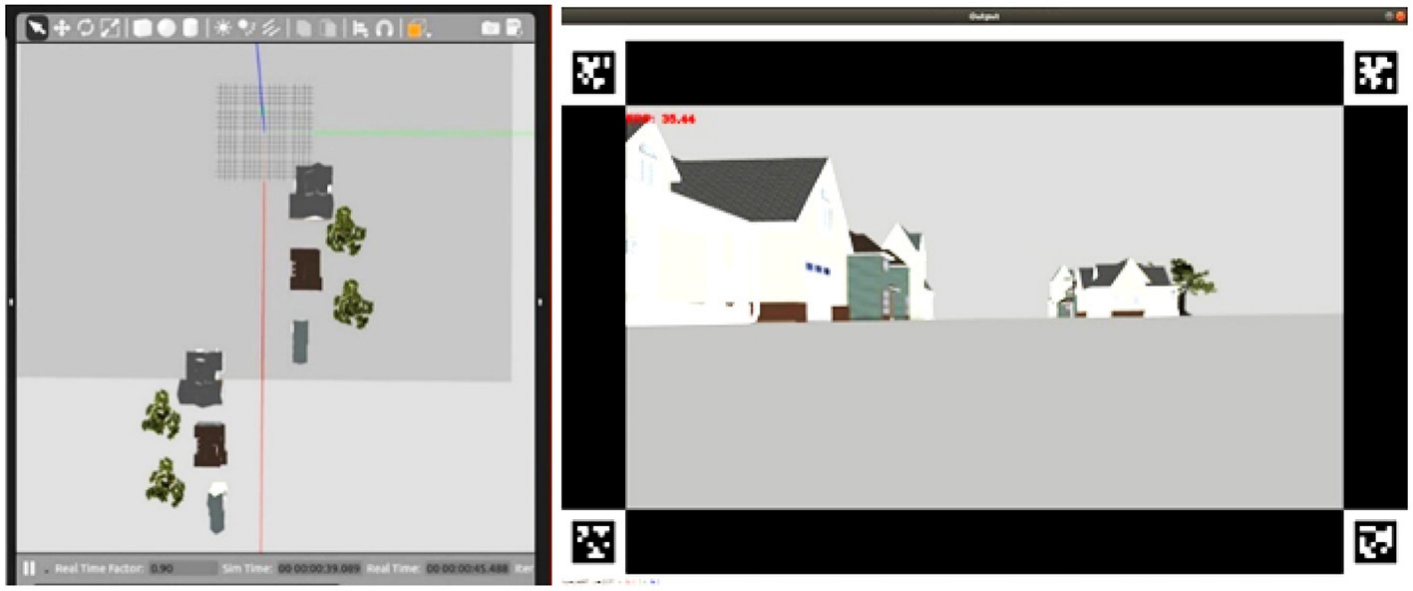
Exploration task. **Left**: top view of the simulated environment used in the test allowing the experimenters to visualize the whole set-up off-line. **Right**: first-person view seen by the user.

In the Exploration test, participants explored a straight path flanked by houses. The environment consisted of six houses and four trees (Figure 7). The task required the participants to drive the drone forward along the path and control the drone's gaze to identify the possible presence of trees between the alleys separating the houses. In particular, the participants had to take off, move forward until they reached the first alley, direct their gaze to it to identify the possible presence of a tree, redirect the gaze toward the straight path, and repeat these operations with all the alleys encountered along the way. Once they had inspected all the alleys and reached the end of the route, they could land. The system calculated the time from take-off to landing of the drone and took a measure of performance.

**Figure 8:**
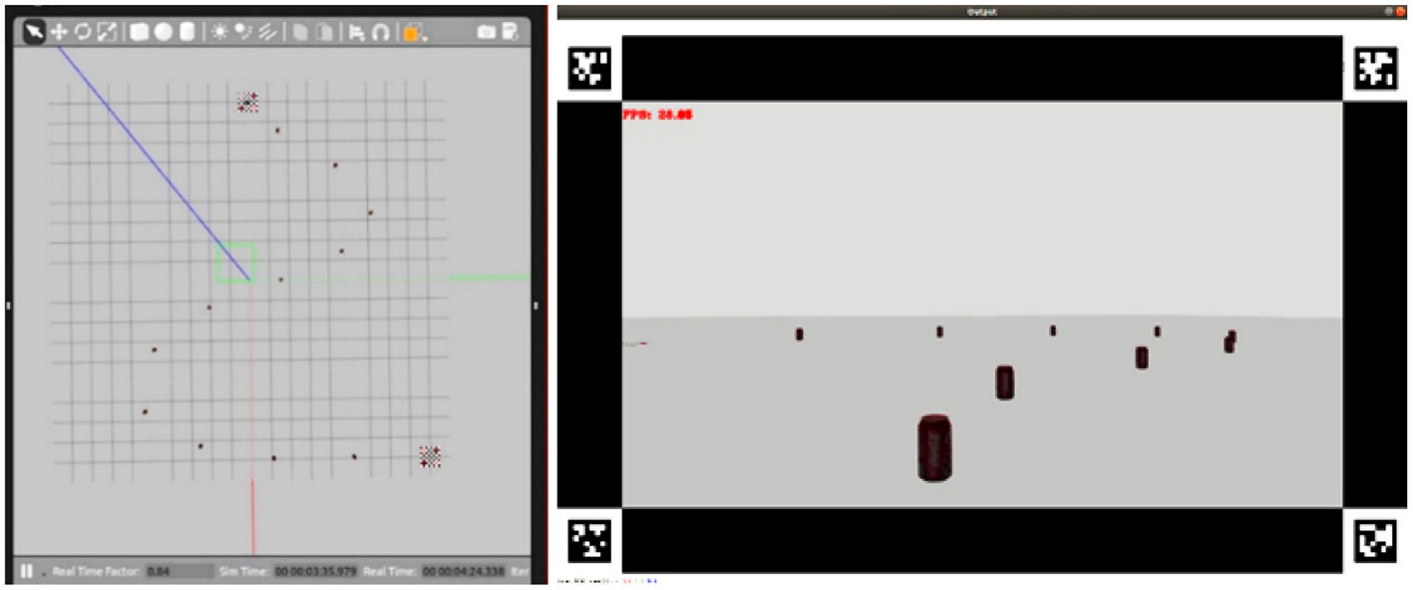
Accuracy task. **Left**: top view of the simulated environment used in the test allowing the experimenters to visualize the whole set-up off-line. **Right**: first-person view seen by the user.

In the Accuracy task, the environment involved a sequence of beacons located on the floor to form a track (Figure 8). The participants had to follow the track as fast and precisely as possible. The average distance of the drone from the closer beacon (accuracy), and the time taken to reach the end of the track (efficiency), were used as a measure of performance.

The closest position of the drone to a certain beacon was used to calculate their distance:


$$\begin{array}{l} \text{​}d(beacon,drone)\\ \;=min_{(x,y)drone}\sqrt{{\left(x_{beacon}-x_{drone}\right)}^{2}+{\left(y_{beacon}-y_{drone}\right)}^{2}} \end{array}$$

Then, the average distance of the drone from the track is computed as the average of the distances of the drone from the set B of beacons on the track


$$\begin{array}{l} \mu_{d}=\underset{beacon\epsilon B}{\sum }\;\frac{d\left(beacon,\;drone\right)}{\left|B\right|} \end{array}$$

where B is the set of considered beacons.

### Questionnaires

A background survey was administered at the beginning of the experiment, including both demographic questions and questions related to previous relevant experiences. This questionnaire was intended to identify possible correlations between the participants' traits and previous experiences and the performance and experience in the experiments. Two additional questionnaires were administered after each session of the experiment: one for the perceived embodiment of the device and one concerning the quality of the virtual experience.

The first questionnaire on perceived embodiment was composed of 20 statements taken from previous Embodied questionnaires (Casper et al., 2015; Roth and Latoschik, 2020; Peck and Gonzalez-Franco, 2021) or specifically adapted or created for the current experiment. Participants were asked to assess how much they agreed with the statements on a 10-point Likert scale (1: completely disagree; 10: completely agree). The aim of the questionnaire was to investigate various aspects of the sense of embodiment intended as the perception of one's physical body while interacting with virtual reality through the NUI or traditional interface. The statements in particular aimed at assessing these three aspects of embodiment: (1) Self-location: perception and localization of somatic stimuli, statements 6, 7, 9, 14, 15, 16, 20; (2) Body-ownership: awareness of ownership of the body, statements 8, 11, 12, 13, 15, 16, 17, 18, 19; (3) Agency: sense of control over movements, statements 2, 3, 4, 5, 10. The items of the questionnaire on embodiment are reported in Table 1.

**Table 1:** Items of the embodiment questionnaire used in this study.

**1**	**I was piloting the drone**
**2**	**The drone responded to my commands**
**3**	**The drone moved exactly as I wanted it to**
**4**	**I felt that I was responsible for the movements I saw**
**5**	**The drone was moving on its own**
**6**	**I felt like I was in the virtual environment**
**7**	**It was as if my eyes were the eyes of the drone**
**8**	**I felt like the drone**
**9**	**I had the feeling that I was moving in sync with the drone**
**10**	**It felt like my hands were in control of the drone**.
**11**	**I felt like I was outside my body**
**12**	**I felt like I had another body**
**13**	**I had the feeling that the drone's movements were affecting my movements**
**14**	**During the take-off of the drone I felt a sense of elevation**
**15**	**I felt like I was floating like a drone**
**16**	**I felt a sense of lowering during the drone's landing**
**17**	**I felt that I was, in some way, connected to the drone**
**18**	**I had the feeling that the drone was part of me**
**19**	**I had the feeling that the drone and I were the same thing**
**20**	**I had the feeling that I was in the drone's place**

The Virtual experience questionnaire was based on the SUXES evaluation method (Turunen et al., 2009). Participants rated 8 features of the system on a 7-point Likert scale (1: completely disagree; 7 completely agree). The target of the questionnaire was different for the two sessions of the experiment: the system and keyboard were assessed after condition T, whereas the system, eye-tracker, and hand-control were assessed after the N session. The 8 items evaluated by the Virtual experience questionnaire are reported in Table 2.

**Table 2:** Items of the SUXES Virtual experience questionnaire used in this study.

1 Is fast
2 Is pleasant
3 Is simple
4 Has no error
5 Is easy to learn
6 Is natural
7 Is useful
8 I would like to use it again

### Procedures

**Figure 9:**
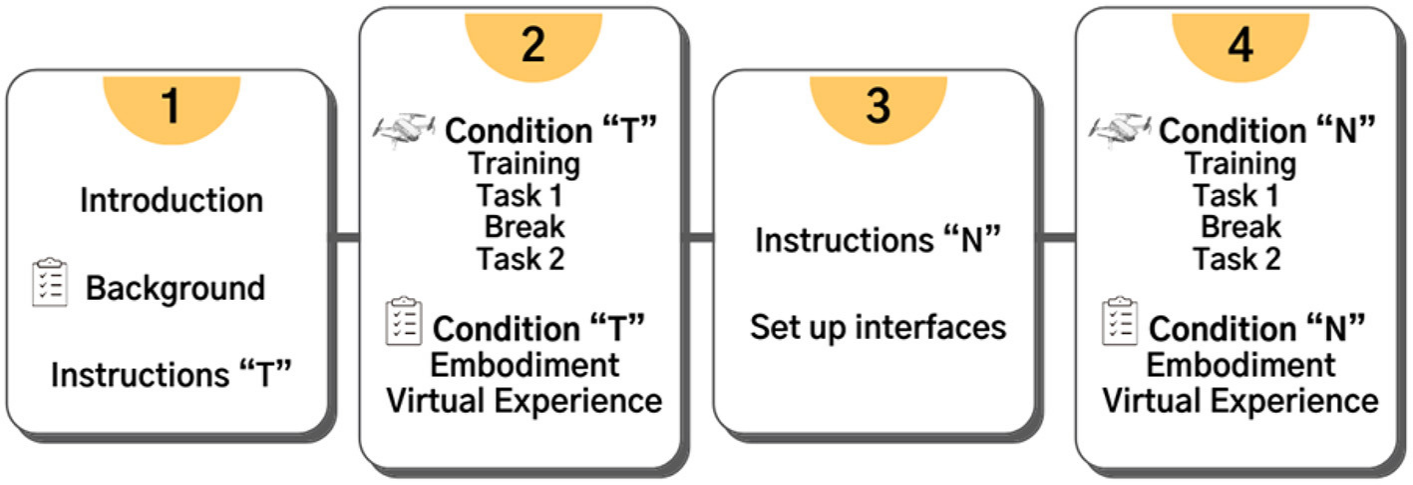
The four phases of the experiment. The order of the conditions T and N (and the related instructions preceding them) were differentiated for the two groups of participants.

The whole experiment lasted about 1 hour. The experiment was organized into four phases (Figure 9), the succession of which depended on the order of the two conditions/tasks. To facilitate the explanation of the structure of the experiment to the users, they were asked to watch a series of introductory slides, and an example video of the tasks. Once the basic concepts were understood, the questionnaire on previous experience was administered, and an identification code was assigned to the participant to guarantee anonymity. The instructions for the commands and execution of the task were then shown depending on the first task to face.

The N condition required additional settings. For the hand-control, participants were asked to wear the light blue glove with their right hand. The webcam's view was shown to allow the participant to understand the field of action within which they could move their hand to issue commands to the drone. The Pupil Core goggles were then worn by the participant, and the experimenter tuned the position and direction of the internal cameras to correctly capture the eye gaze. The participant then assumed a comfortable position to view the screen through the front camera of the Pupil Core. Depending on the height of the user, thicknesses were placed under the screen to avoid unnatural postures. Then the calibration procedure was carried out. To this purpose, while keeping the head still, the participant directed the gaze to seven target stimuli appearing in sequence on the screen.

For both conditions, after reading the instructions of the commands and of the first task (and performing the necessary procedures, in particular in condition N) the participant underwent a training phase to allow the familiarization with the interface and the drone commands. In the virtual environment of the first task, the participant was free to move and test the system for 2 min in the T condition and 3 min in the N condition; in particular, in the latter condition the participant was supported by the experimenter who gave indications to understand in detail the commands issued with the Pupil Core and those with the hand gestures. At the end of the training, the actual experimental phase was carried out on the first task, and this was recorded. After a short break, the instructions for the second task were shown, which was then carried out and recorded. The phase two of the experiment ended with the administration of the two questionnaires (virtual experience and embodied cognition). In phase three, the procedure was repeated, the participant read the instructions of the next condition, performed the respective training and tasks, and then answered the two questionnaires.

### Data Analysis

We carried out data analysis using the statistical open-source software “R”, version 3.6.3. We considered two performance metrics: (a) time of execution in both tasks; (b) accuracy (average distance from the closer beacon) for the second task; (c) frequency distributions for the two tasks. We performed Kernel Density Estimation (KDE) to compare the frequency distribution over the two conditions. To assess the statistical significance of the results we used t-tests for paired samples. To detect possible correlations with the characteristics of the participants, obtained from the questionnaire on previous experiences, the participants belonging to the first quartile for each condition were also analyzed separately. The same was done with the scores obtained through the “Embodied cognition” and “Virtual experience” questionnaires.

## Results

Overall, the results confirmed only one out of three hypotheses. In particular, the Keyboard traditional control proved to lead to a higher performance than the NUI, thus falsifying our first hypothesis. Instead, in agreement with the second hypothesis, the degree of embodied cognition assessed by the questionnaires showed a higher score with the NUI. Finally, the analyses based on the first quartile of the questionnaires found no relevant correlations in terms of either sample characteristics or level of embodiment, so disconfirming the third hypothesis.

### Previous Experience of Participants

Data from the questionnaire showed that 83.1% of participants had never used a drone before. The sample was thus divided into 61% who had had previous experience with video games and 39% with little or no experience. Familiarity with the use of keyboards was reported by 47.5% of the participants. None of the participants had had previous experience with NUIs similar to the one employed in the test. Experience in video games and keyboard proved to lead to a better performance in condition T; no correlation was found with results in N. Experience gap was not related to a better performance with natural interfaces. As shown in the results both groups, independently of experience, had a higher performance in condition T.

### Performance

**Figure 10:**
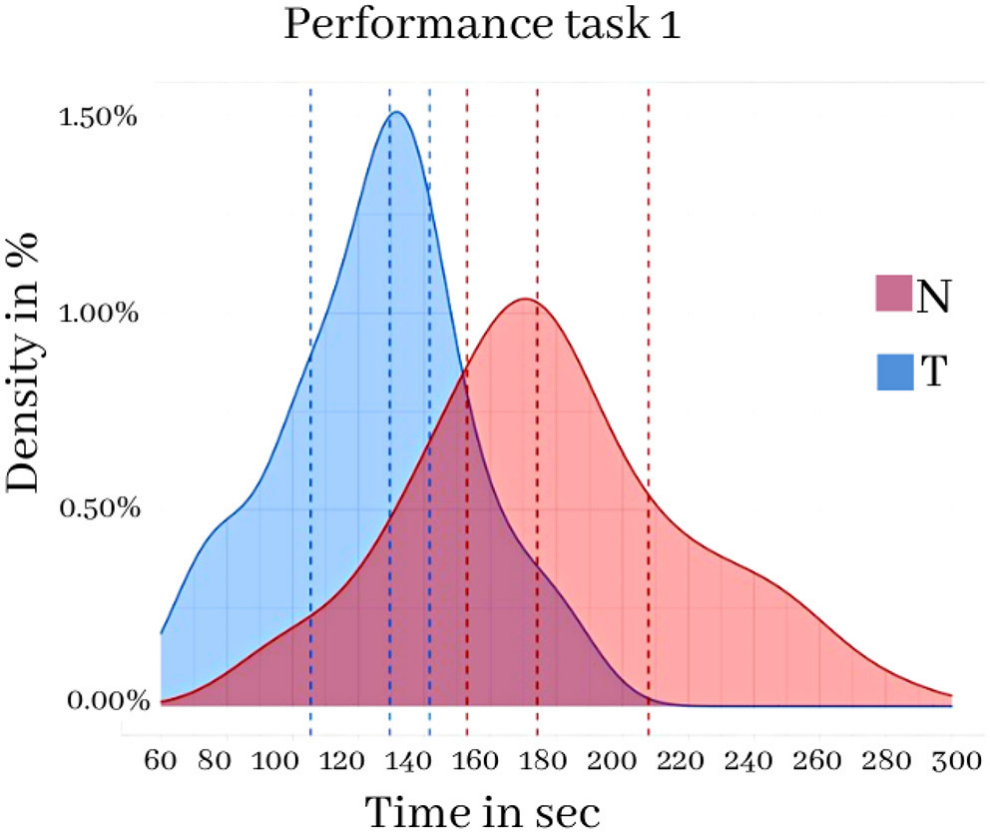
Exploration task 1 in the two conditions N and T. Frequency distribution of the time taken to complete the task in the two conditions N and T. Vertical lines indicate the first, second and third quartiles (25%, 50%, 75%).

Figure 10, related to the performance (completion time) in the Exploration, shows that the time employed by the participants to complete the path was longer for the N condition (red) than for the T condition (blue) with a mean difference of 61.73 seconds (t = −8.04, ds = 7.67, *p* < 0.000).

**Figure 11:**
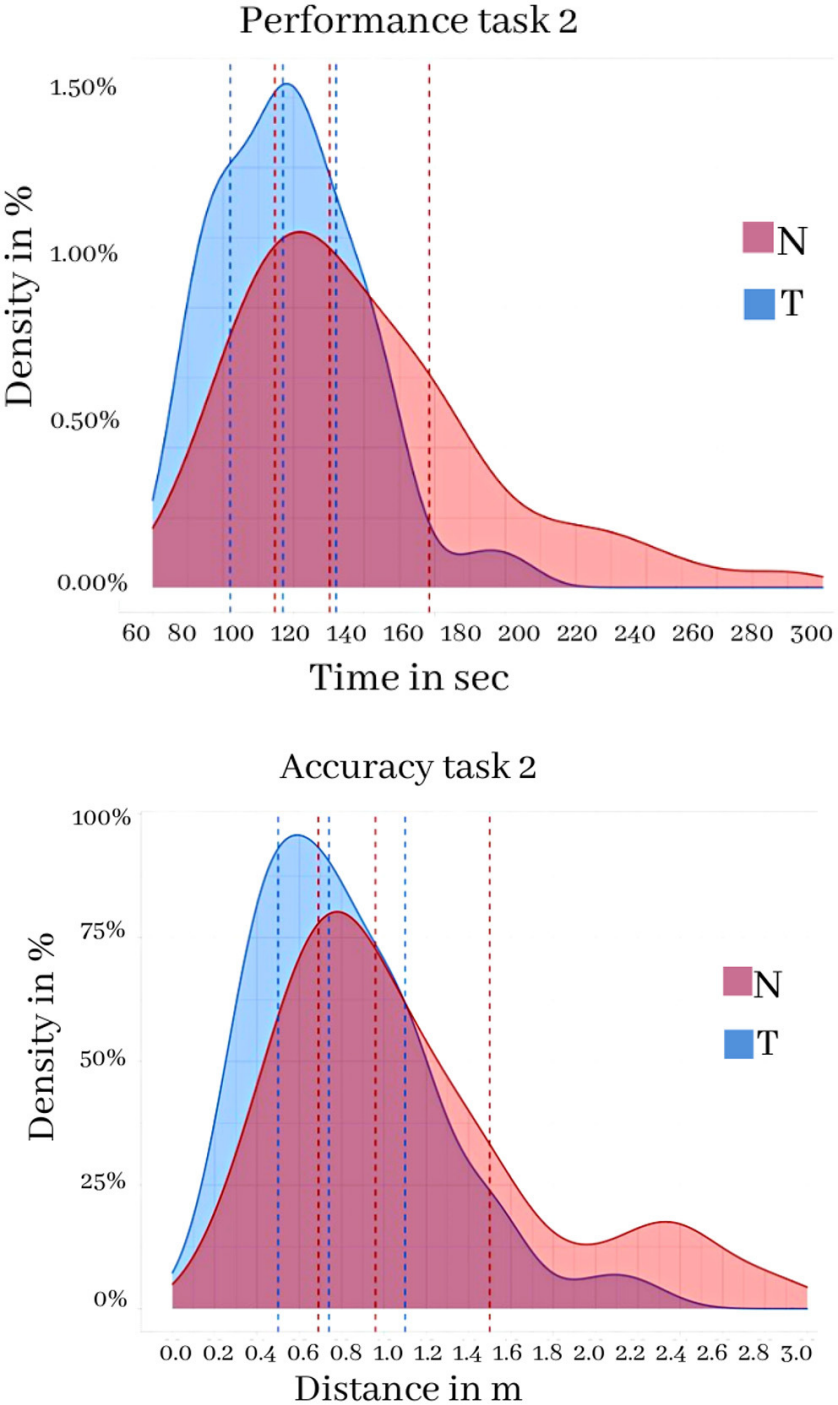
Accuracy task 2 in the two conditions N and T. Top: performance measured as task execution time. Bottom: accuracy as average distance from the beacons. Vertical lines indicate the first, second and third quartiles (25, 50, 75%).

Figure 11, referring to the performance (completion time) and accuracy (average distance from the closest beacon) related to the Accuracy task, shows the frequency distribution relating to such metrics. The results show an average difference of 23.68 s in the execution time (*t* = −4.27, ds = 5.54, *p* < 0.000) and an average distance of 0.34 m to the beacons (*t* = −2.80, ds = 0.12, *p* < 0.001).

The alternation between the two possible orders of the two experiments was done to avoid affecting the results. Afterwards, an analysis was carried out to exclude the influence of one type of condition over the second one. In particular, we compared the two possible orders of the experiments with a t-test that showed no significant difference in mean performance (t = 0,84, *P* > 0.05). The order of the experiments did not significantly affect the outcome possibly because there was not a significant learning effect of the knowledge acquired with one experiment over the other.

### Virtual Experience

**Figure 12:**
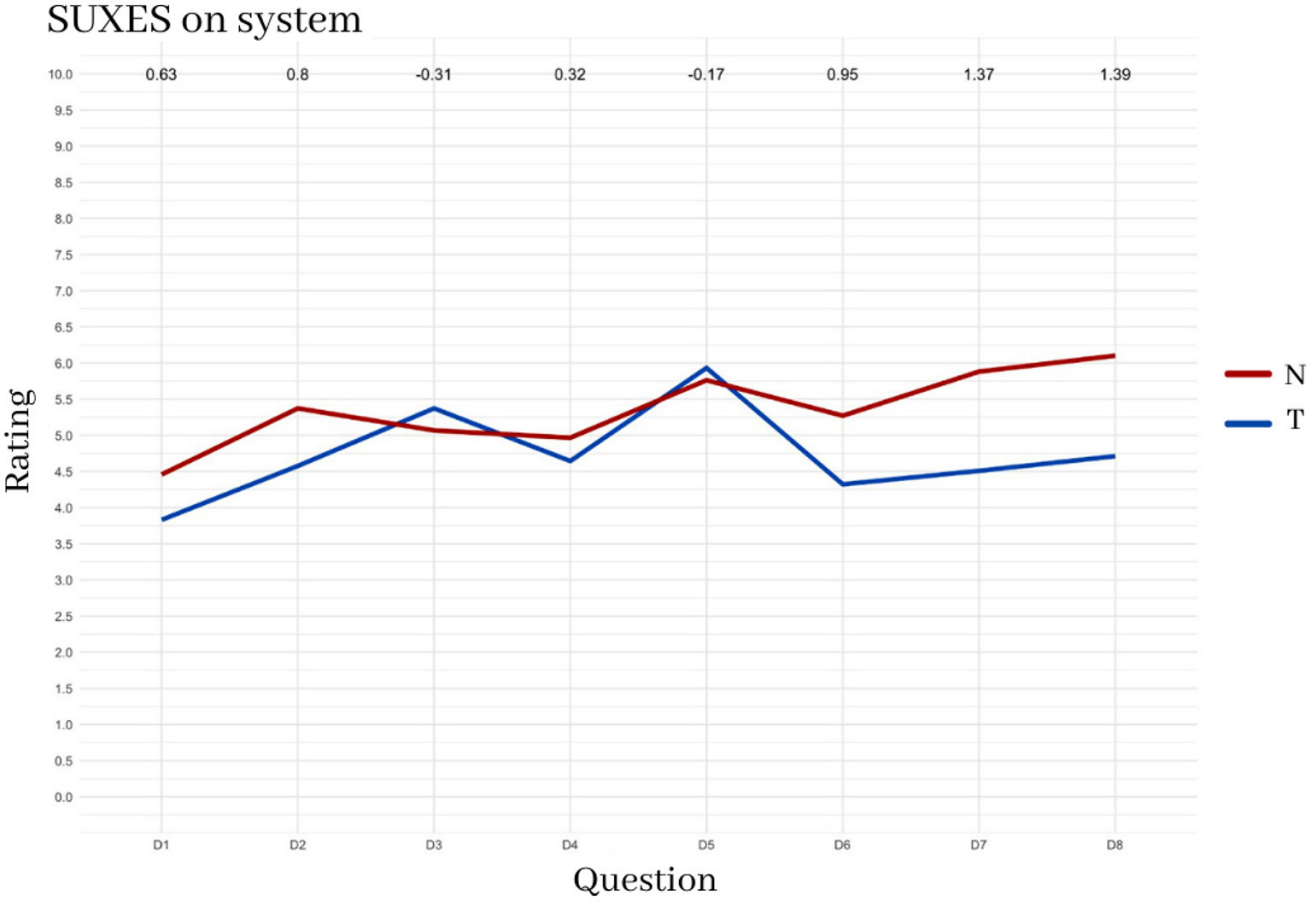
System-related answers of the SUXES questionnaire in the two conditions N and T.

Figure 12 shows the results of the SUXES scores for the two conditions, indicating no relevant differences. The T system obtained an average rating of 4.70 (sd = 1.77); the N system obtained an average rating of 5.29 (sd = 1.49). The T condition scored slightly higher than N for items 3 and 5 (“is simple”; “is easy to learn”), thus suggesting that the use of the keyboard is easier.

**Figure 13:**
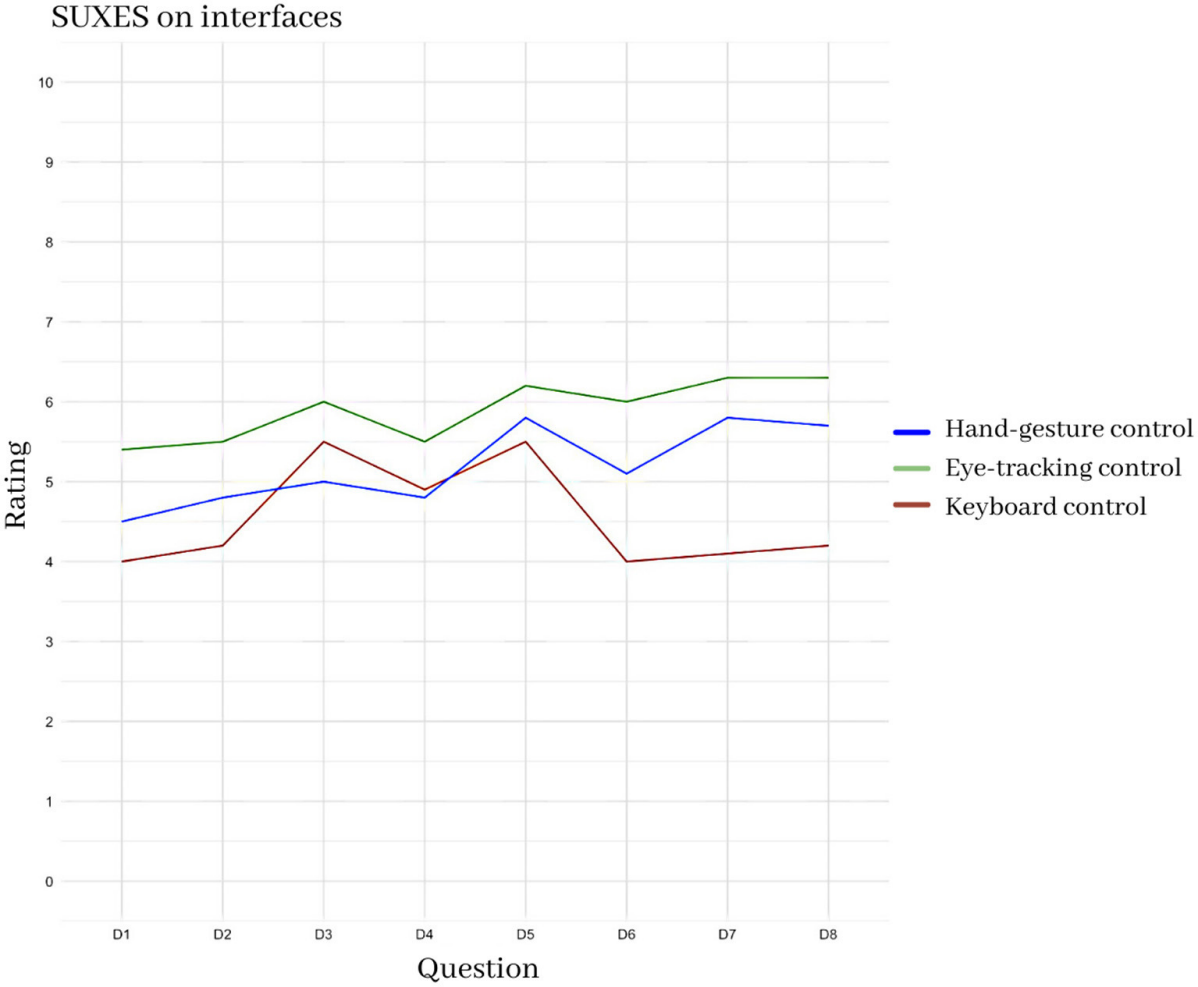
Comparison of the average scores for the SUXES questionnaire for the different interfaces, and interface components: keyboard, eye-control, hand control.

Figure 13 instead shows the SUXES scores for the three different interfaces or interface components: the keyboard, the eye-tracking control, and the hand-gesture control. All of them were analyzed separately with separete SUXES. Keyboard obtained an average rating of 4.57 (sd = 1.79); eye-tracking control obtained an average rating of 5.83 (sd = 1.28); hand control obtained an average rating of 5.11 (sd = 1.50).

The results show that the eye control resulted to be better than the others in each aspect.

### Embodied Cognition

**Figure 14:**
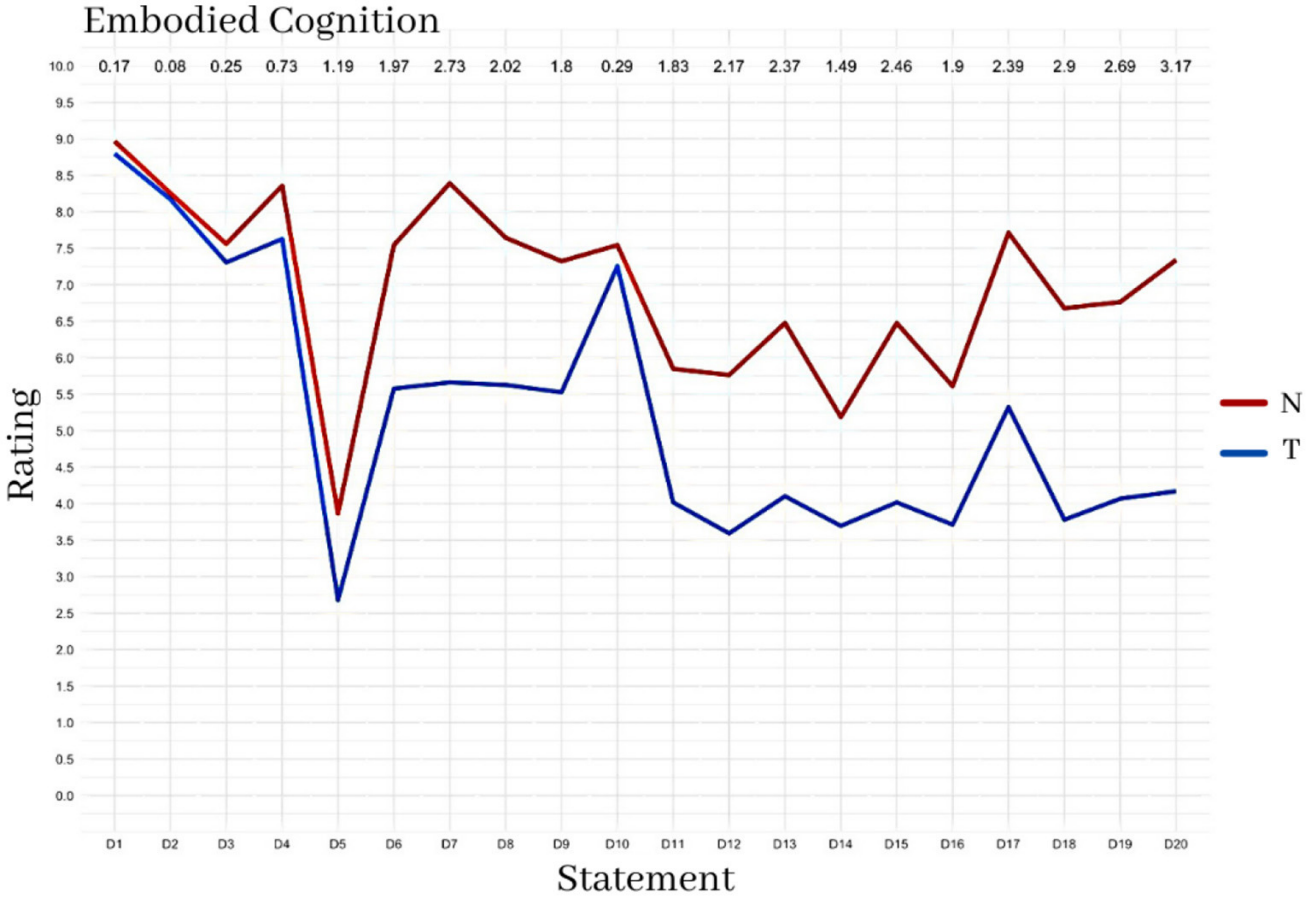
Average response scores to the Embodied Cognition questionnaire.

Figure 14 reports a summary of the answers to the questionnaire on Embodied cognition. The results show a higher score for the N condition than for the T condition. The answers that differ most in the two conditions are those related to the sense of “self-location” (items: 7-9-15-20) and “ownership” (items: 8-13-15-19).

## Discussion

The results obtained, although partially in disagreement with our initial hypotheses, reveal interesting effects of the two interfaces. Despite only half of the participants being familiar with the keyboard, this proved to be a more efficient interface than the others in terms of the user's performance. Several aspects could explain this result. First, regardless of experience, everyone knows how to use a keyboard. Conversely, the use of NUI, although natural, requires learning as it is something new to the participants, as suggested by the SUXES questionnaire items related to learning. In this respect, the difference of performance of N versus T that decreases substantially by passing from the first Exploration task to the second Accuracy task (it is worth reminding that the two tasks were always presented in the same order with all participants) suggests that learning can improve the usability and performance of the NUI interface. This effect could be however also due to a difference in the two tasks. For instance, the NUI seems better suited for the Accuracy task where the participants have to dynamically control the drone flying along a predefined track as fast and accurately as possible, a challenge for which the eye-tracking and hand-gesture interfaces might be particularly well suited. Further investigations are needed to ascertain these two possibilities.

Another possible explanation of the lower performance of the NUI with respect to the traditional interface is suggested by the participants' Virtual-experience ratings related to the two interfaces. Indeed, the results show that the eye-tracker control was rated as the best, but it was combined with the hand-gesture control that received a lower score and this could have overall lowered the performance. Future work could thus investigate possible alternatives to the gesture control.

Considering the results of the Embodiment questionnaire, and specifically in relation to the “Sense of agency”, the NUI did not perform well as expected. The discretization of actions (button clicked/not clicked) via the keyboard is different from the continuous command given by the eyes and the hand, which is extremely sensitive. In particular, some participants may have encountered problems maintaining a fixed point on the screen due to involuntary movements and saccades. Moreover, hand control requires high levels of proprioception to perform an accurate control, while participants often found themselves giving unintentional commands as they were unaware of the exact position of their hand in relation to the webcam lens.

These aspects led us to reconsider the actual naturalness of the commands used. Keeping the head constantly still and intentionally directing the gaze without error is not a natural behavior in humans. For example, instinctively looking at a point to the right side of the visual field involves moving the head to that side and not simply directing the gaze to it. Moreover, the position used to “drive” the drone through the hand-gesture control is something new to which participants were not accustomed. Considering that cars are common tools for humans, they perceive as “natural” the control of a moving devices through a steering wheel, so the hand-gesture control used in the experiment could appear non natural.

In summary, there are several factors that might have contributed to higher performance of the traditional interface versus the NUI: (a) prior knowledge of the keyboard, regardless of familiarity; (b) association of an efficient interface (eye-tracking) with a less efficient one (hand-control); (c) high sensitivity of the NUI devices; (d) difference between discrete commands (in T) and continuous commands (in N); (e) lack of naturalness of some aspects of the controls used in the condition N.

Overall, the results achieved in the presented experiments seem to testify that, more than ten years after the Norman's claim that “*natural interfaces are not natural*” (Norman, 2010), and some critical analysis of the related problems (see for example Malizia and Bellucci, 2012), much research is still needed to model and design fully efficient, effective and satisfying NUIs according to the familiar definition of usability (Bevan et al., 1991; Bevan, 1995). This calls for taking into account *the users*, to create a system adaptable to their technical skills and physical abilities; *the application*, to choose the best interaction channels for each intended task; *the available equipment*, to take into account the current limitations and possibly create pluggable modules to update following future technological advances; and *the environment where the application is deployed*, to anticipate and overcome possible obstacles created by environmental conditions, as for example light level and noisy background for gesture recognition.

### Limitations and Future Work

The results of the experiments highlight important weaknesses in the proposed system that prompt various directions for future investigation. Numerous modifications could optimize the controls that we started to investigate here and demonstrate a real advantage of natural interfaces over traditional ones. Regarding eye control, an improvement might involve the possibility of moving the head and not only the eyes. Regarding the hand control, this should more closely reflect the naturalness of common driving gestures that are experienced by humans. Moreover, the use of the glove as an intrusive element should be eliminated. The sensitivity of both controls should be modified so that unintentional changes are less impairing. Testing the eye-tracker and hand-gesture devices of the N condition in isolation could allow the evaluation of their distinct efficiency.

To increase the level of embodiment, it could be useful to implement sounds coming from the device and to implement voice control. Another important possibility is that the utility of the NUIs would manifest more strongly when one has to control a higher number of degrees of freedom of the drone, for example the drone flight altitude and the zooming in and out of the camera. It would also be interesting to test the interfaces when the participants control real drones in real environments.

In the light of the lower performance found with the NUI continuous control in comparison to the traditional interface based on discrete commands, and also based on its higher embodiment sense, future work might also compare the two interfaces in different experimental conditions to better understand if in other conditions NUI leads to a higher performance. In particular, the NUI might yield a higher performance when the control becomes more challenging, for example if the drone is perturbed by random external forces for example simulating a flight with strong/irregular wind. Indeed here an embodied natural control might result more automatic (vs. deliberative), hence freeing the pilot's higher level cognitive functions to facilitate the drone control.

In addition, the continuous nature of the NUI with respect to the discrete one of the traditional interfaces might facilitate a fine-tuned control. To test this effect, one might also consider a third experimental condition where the goal of the pilot is to keep the drone as stable as possible in a certain area in the face of the perturbations described above. This would allow one to collect a very informative score of the continuous/discrete features of the two interfaces.

A further interesting test could be directed to compare the two interfaces with two participant groups, one formed by drone flight expert pilots and a second one by naive participants. This could give valuable information about the potential utility of the NUI with respect to the traditional control. In particular, it could for example reveal that the NUI only benefits the naive group, or that, more interesting, it benefits both groups.

Finally, testing participants with physical impairments could be more significant as the success of Assistive Technology is defined and determined by people's possibilities, perspectives, and goals. For example the interfaces proposed here might be very useful for people with arm and hand limitations that make it difficult for them to control devices via a keyboard.

## Conclusions

A shift from traditional interfaces toward the so-called Natural User Interfaces (NUIs) has been predicted for many years. However, the keyboard is a tool that has been successfully in use for more than a century. Eradicating such a long-lived tool from everyday life is not easy and would require a major improvement. This has in particular been shown in this study, suggesting that effective NUIs leading to a higher performance than a keyboard-based interface requires notable advancements. In particular, traditional interfaces are “artificial” but at the same time very accurate. Thus, to overcome their performance the usability of NUIs has to go above a certain threshold that compensates for such accuracy.

The study highlighted several weaknesses and limitations for the NUI proposed here that are presumably responsible for not confirming the initial hypotheses on higher performance of the employed NUIs with respect to the traditional interface. Working on that could be the next step. Natural interfaces have great potential, in particular for Assistive Technologies, but this study shows that their actual utility depends on the possibility of achieving a strong naturalness to compete with more traditional interfaces that are commonly used by humans.

## Data availability statement

The raw data supporting the results of this article can be publicly downloaded from the website: https://osf.io/cz6aq/.

## Ethics statement

The studies involving human participants were reviewed and approved by Ethics Committee of the Department of Dynamic and Clinical Psychology and Health Studies of Sapienza University of Rome. The patients/participants provided their written informed consent to participate in this study.

## Author contributions

GB conceived the initial idea of the experiment. FP prepared the software to run the experiment. MDV ran the experiment, collected the data, performed the statistical analysis, and wrote the first draft of the manuscript. FP, MDM, AB, and GB contributed to write sections of the manuscript. All authors contributed to the design of the study, data interpretation, and manuscript revision, and approved the submitted version.

## Funding

This research has received funding from the European Union's Horizon 2020 Research and Innovation Program under Grant Agreement No. 713010, Project GOAL-Robots—Goal-based Open-ended Autonomous Learning Robots, and under Grant Agreement No. 952095, and Project IM-TWIN—from Intrinsic Motivations to Transitional Wearable INtelligent companions for autism spectrum disorder. This research has also received support from AI2Life Srl Innovative Startup, a spin-off company of ISTC-CNR, https://ai2life.com/, and from AS-AI 2020-2021—Advanced School of AI, https://as-ai.org/.

## Conflict of Interest

GB was employed by AI2Life Srl Innovative Startup, Spin-Off of ISTC-CNR. The remaining authors declare that the research was conducted in the absence of any commercial or financial relationships that could be construed as a potential conflict of interest.

## Publisher's Note

All claims expressed in this article are solely those of the authors and do not necessarily represent those of their affiliated organizations, or those of the publisher, the editors and the reviewers. Any product that may be evaluated in this article, or claim that may be made by its manufacturer, is not guaranteed or endorsed by the publisher.
